# Insomnia partially mediates the association between ADHD traits and depressive symptoms in Japanese nursing students

**DOI:** 10.1007/s44192-026-00562-w

**Published:** 2026-08-07

**Authors:** Minoru Hattori, Akane Hirohata, Hideki Tanaka, Naoko Hasunuma

**Affiliations:** 1 Center for Medical Education, School of Medicine, Hiroshima University, 1-2-3 Kasumi, Minami-ku, Hiroshima, 734-8553 Japan; 2 Wakaba Ryoikuen, Hiroshima Prefectural Rehabilitation Center, Hiroshima, Japan; 3 Graduate School of Psychological Science, Hiroshima International University, Hiroshima, Japan

## Abstract

Nursing students with attention-deficit/hyperactivity disorder (ADHD) traits may struggle with demanding academic and clinical training, potentially increasing the risk of insomnia and depressive symptoms. This study examined whether insomnia statistically mediates the association between ADHD traits and depressive symptoms in this population. A cross-sectional survey was conducted with 131 nursing students (96.9% female; mean age = 20.4 ± 2.3 years) at two Japanese universities in late October–November 2018, prior to the COVID-19 pandemic. Participants completed measures of ADHD traits (ASRS v1.1 Part A), depressive symptoms (PHQ-9), and insomnia (Athens Insomnia Scale). A bootstrapped mediation analysis (5,000 resamples), adjusted for age, sex, and sleep/circadian factors (sleep duration, chronotype, social jetlag), was performed. ADHD traits showed a weak positive correlation with insomnia (r = 0.29, *p* < 0.001) and a moderate positive correlation with depressive symptoms (r = 0.44, *p* < 0.001); insomnia and depressive symptoms were moderately correlated (r = 0.54, *p* < 0.001). Insomnia partially mediated the association between ADHD traits and depressive symptoms: the indirect effect was significant (β = 0.105, 95% bootstrap CI [0.024, 0.194]; Sobel z = 2.49, *p* = 0.013), accounting for approximately 26% of the total effect, while the direct effect remained significant (β = 0.293, *p* < 0.001). A Monte Carlo post-hoc power analysis indicated that statistical power for the indirect effect was 0.82. These findings, interpreted within the constraints of a cross-sectional design, identify insomnia as a candidate mechanism linking ADHD traits to depressive symptoms and suggest that integrating sleep health screening and targeted sleep-focused interventions into nursing education may offer a feasible and scalable preventive approach to supporting student mental health within higher-education support systems.

## Introduction

Nursing students face increasing academic and emotional demands that compromise performance, well-being, and professional development. A pre-pandemic meta-analysis estimated that 27% of medical and nursing undergraduates report clinically relevant depressive symptoms [1]; more recent evidence suggests that rates have risen further during and after the COVID-19 pandemic [2]. Depression during training is linked to poorer academic outcomes, reduced empathy, more clinical errors, and higher attrition—issues directly affecting patient safety and healthcare quality [3].

Attention-Deficit/Hyperactivity Disorder (ADHD) is a prevalent neurodevelopmental disorder, affecting approximately 5–7% of the population [4]. These traits are often overlooked in adults but are associated with burnout [5], emotional dysregulation [6], and diminished clinical performance. Students with prominent inattention symptoms at university entry often struggle academically over time. The repeated failures and criticism stemming from ADHD-related difficulties can erode self-esteem and overall well-being [7, 8]. University students with ADHD reported significantly more anxiety and depression than those without [9]. In nursing, which involves high-stakes placements, multitasking, and emotional labor, students with ADHD traits may be particularly vulnerable to distress [10].

Increasingly, poor sleep is recognized not merely as a symptom but as a contributing factor in mental health decline, particularly among individuals with neurodevelopmental vulnerabilities [11]. A landmark randomized controlled trial with mediation analysis demonstrated that improving sleep causally reduces depressive and anxiety symptoms in young adults [11], providing direct evidence that sleep is a modifiable upstream contributor to mood. Within healthcare training, sleep disturbance is among the strongest modifiable correlates of depression and burnout in nursing students [12]. Despite this plausible neurobiological pathway, the mediating role of insomnia between ADHD traits and depressive symptoms remains underexplored in pre-licensure nursing students. While prior research has established bivariate associations among ADHD symptoms, sleep disturbances, and mood problems in undergraduates [13], formal mediation analyses have largely focused on adult community samples or on practicing healthcare professionals [14]. Pre-licensure students have rarely been the focus, despite being the developmental window in which preventive interventions are most feasible.

Nursing education is a particularly informative context in which to examine this pathway. Early-morning clinical placements, irregular ward rotations, and high-stakes performance evaluation place sustained pressure on the same circadian and arousal systems that are already implicated in adult ADHD. We therefore hypothesized that insomnia symptoms would partially account for the cross-sectional association between ADHD traits and depressive symptoms in Japanese undergraduate nursing students, and that quantifying this indirect pathway would identify a tractable, education-embedded target for prevention. By clarifying this association, we aim to inform sleep-focused, low-intensity supports for students at risk and to contribute to preventive student-mental-health strategies in nursing education.

## Methods

### Participants and study design

In total, 131 nursing students from two participating universities were included in this cross-sectional study. The study was observational in nature; the sample size was not based on a specific power calculation, and we aimed to include as many eligible students as possible. A paper-based survey was used. After the researchers explained the study in writing and verbally, participants completed self-report scales anonymously. The survey was administered only to students who were willing to participate and was distributed at the end of class sessions. Data collection was conducted between late October and November 2018, immediately following institutional ethical approval on October 26, 2018 (see Sect. 2.2), and was completed prior to the COVID-19 pandemic.

Publication was delayed by pandemic-related disruptions to academic workloads. We therefore present these findings as a pre-pandemic baseline characterizing the relationship between ADHD traits, insomnia, and depressive symptoms in Japanese nursing students under routine educational conditions. This baseline is methodologically valuable because it is uncontaminated by pandemic-related disruptions to sleep, social rhythms, and clinical training. It may serve as a reference point against which post-pandemic cohorts, which show substantially higher rates of psychological distress in nursing students [2], can be compared.

### Ethical considerations

This study was approved by the Institutional Review Board of Hiroshima University School of Medicine (Approval No. E-1408; approved on October 26, 2018) and was conducted in accordance with the Declaration of Helsinki. The approval immediately preceded the start of data collection in late October 2018. Written and verbal informed consent was obtained from all participants. To protect privacy and confidentiality, all data were anonymized.

### Measures

ADHD traits were assessed using the Japanese version of the Adult ADHD Self-Report Scale (ASRS v1.1 Part A), a six-item screening tool developed by the World Health Organization and a panel of ADHD experts [15, 16]. These six items are considered the most predictive of clinically significant ADHD symptoms. Each item was scored on a 0–4 Likert scale, yielding total scores ranging from 0 to 24. Internal consistency for the ASRS-6, computed on the same Likert scoring used in all analyses, was Cronbach’s α = 0.677 (95% CI 0.584–0.756) and McDonald’s ω = 0.679. These values are comparable to those reported for the Japanese ASRS in undergraduate samples [15]. Exploratory factor analysis confirmed the expected two-factor (inattention/hyperactivity) structure (eigenvalues 2.38 and 1.35), with subscale α values of 0.721 (inattention, items 1–4) and 0.662 (hyperactivity, items 5–6). The mean inter-item correlation (0.27) was within the ideal 0.15–0.50 range recommended for short multidimensional scales.

Depression was assessed using the Japanese version of the Patient Health Questionnaire-9 (PHQ-9), with total scores ranging from 0 to 27 [17]. Internal consistency in this study was acceptable (α = 0.766, 95% CI 0.701–0.822).

The Japanese version of the Athens Insomnia Scale (AIS-J) was used to assess sleep disturbance [18]. The AIS consists of 8 items scored from 0 to 3, yielding total scores from 0 to 24, with higher scores indicating greater severity of insomnia symptoms. Internal consistency in this sample was α = 0.724 (95% CI 0.645–0.791). Prior studies support its validity in Japanese populations [18].

Sleep patterns, chronotype, and social jetlag.

Participants reported their usual bedtimes and wake-up times for both weekdays and weekends. Chronotype was calculated using the sleep-corrected midpoint of sleep on free days (MSFsc), which accounts for potential sleep debt accumulated during weekdays. Social jetlag (SJL) was defined as the absolute difference between the mid-sleep point on free days and workdays (|MSF − MSW|), allowing assessment of the extent of circadian misalignment. All indicators were calculated in accordance with established chronobiological methods [19].

### Statistical analysis

Descriptive statistics were computed for all variables. Continuous variables are reported as means and standard deviations (SD), and categorical variables as frequencies and percentages. Missing data were minimal (< 2%) and handled using listwise deletion; BMI had 8% missingness and was therefore excluded from the primary mediation model and examined in a sensitivity analysis with mean imputation.

Group differences between participants with and without ADHD traits were assessed using independent-samples t-tests for continuous variables and Fisher’s exact tests for categorical variables. Effect sizes were interpreted using Cohen’s d and Cramér’s V, respectively. Pearson correlations were calculated to examine bivariate associations among ADHD traits, insomnia, depression, and related variables. Following conventional benchmarks, correlations of |r|< 0.30 were described as weak, 0.30 ≤|r|< 0.70 as moderate, and |r|≥ 0.70 as strong.

Mediation analysis was conducted using SPSS v28 with the PROCESS macro (v4.2, Model 4) [20] to test whether insomnia severity (AIS total score) mediated the association between ADHD traits (ASRS total score) and depressive symptoms (PHQ-9 total score). Indirect effects were estimated using 5,000-sample bias-corrected bootstrap 95% confidence intervals. The model adjusted for age, sex, and sleep/circadian factors (weekday and weekend sleep duration, chronotype as MSFsc, and social jetlag). These covariates were included because each is independently associated with both insomnia symptoms and depressive symptoms in young adults and would otherwise confound the a- and b-paths. Importantly, the AIS (the mediator) indexes subjective insomnia complaints, including sleep-onset difficulty, maintenance problems, and daytime dysfunction, whereas the covariates index habitual sleep timing and circadian alignment, which are conceptually distinct. Adjusting for them therefore isolates the specific mediating contribution of insomnia symptoms over and above general sleep duration and circadian misalignment. A two-sided *p* < 0.05 was considered statistically significant.

To assess the robustness of the mediation result, we conducted three sensitivity analyses: (i) re-estimating the mediation model with the dichotomous (0/1) ASRS endorsement scoring; (ii) re-estimating it using only the inattention subscale (items 1–4); and (iii) re-estimating the primary model with mean-imputed BMI as an additional covariate. We also computed disattenuated correlations using the Spearman correction, given that measurement unreliability attenuates rather than inflates association estimates.

A Monte Carlo post-hoc power analysis [21] was conducted for the indirect effect using the observed standardized a-path (β = 0.253, SE = 0.091) and b-path (β = 0.416, SE = 0.075), with 1,000 simulated datasets and 2,000 bootstrap replications per dataset. With n = 129, statistical power for the indirect effect at α = 0.05 was 0.82, exceeding the conventional 0.80 threshold. Power for the bivariate correlations exceeded 0.92 in all cases.

## Results

### Participant characteristics

Table 1 presents the demographic and clinical profiles of the 131 nursing students (96.9% female, mean age = 20.4 ± 2.3 years). Mean weekday and weekend sleep durations were 6.09 ± 1.00 h and 7.90 ± 1.40 h, respectively. Average scores on key instruments were as follows: ASRS = 9.73 ± 3.24, AIS = 3.93 ± 2.75, and PHQ-9 = 4.77 ± 3.65. Based on recommended cut-off scores, 46.6% met the criteria for at least mild insomnia (AIS ≥ 6), and 48.1% for at least mild depressive symptoms (PHQ-9 ≥ 5). Approximately 11% screened positive for clinically relevant ADHD traits (ASRS Likert sum ≥ 14; n = 14).

**Table 1:** Population characteristics (n = 131)

Characteristic	Total (n = 131)
Age, years	20.4 (2.3)
*Gender, n (%)*	
Female	127 (96.9%)
Male	4 (3.1%)
BMI, kg/m^2^	20.0 (1.9)
Sleep time (weekday), hours	6.09 (1.0)
Sleep time (weekend), hours	7.90 (1.4)
Chronotype (MSFsc), decimal hours	4.21 (0.79)
*Chronotype category, n* (%)	
Morning type	58 (44.3%)
Intermediate type	51 (38.9%)
Evening type	21 (16.0%)
Missing	1 (0.8%)
Social jetlag, hours	0.95 (0.69)
ASRS total score	9.73 (3.24)
*ASRS classification, n* (%)	
0	66 (50.4%)
1	51 (38.9%)
2	11 (8.4%)
3	3 (2.3%)
AIS total score	3.93 (2.75)
*AIS classification, n* (%)	
Minimal (0–5)	70 (53.4%)
Mild (6–9)	31 (23.7%)
Moderate (10–15)	26 (19.8%)
Severe (16–24)	4 (3.1%)
PHQ-9 total score	4.77 (3.65)
*PHQ-9 classification, n* (%)	
Minimal (0–4)	68 (51.9%)
Mild (5–9)	50 (38.2%)
Moderate (10–14)	11 (8.4%)
Moderate to severe (15–19)	2 (1.5%)
Severe (20–27)	0 (0%)

BMI: Body Mass Index; MSFsc: Mid-Sleep on Free Days corrected; PHQ-9: Patient Health Questionnaire-9; ASRS: Adult ADHD Self-Report Scale v1.1; AIS: Athens Insomnia Scale

### Group comparisons

Compared with the negative-screen group, students screening positive for ADHD traits reported significantly higher insomnia severity (AIS: 5.64 ± 2.47 vs. 3.73 ± 2.72; *p* = 0.013; Cohen’s d = 0.71) and depressive symptoms (PHQ-9: 8.07 ± 4.68 vs. 4.38 ± 3.32; *p* < 0.001; Cohen’s d = 1.06), both reflecting medium-to-large effect sizes.

Students screening positive for ADHD traits also exhibited significantly later chronotypes, as measured by corrected mid-sleep time on free days (4.72 ± 0.71 vs. 4.13 ± 0.78; *p* = 0.014; Cohen’s d = 0.77), indicating a delayed circadian phase. No significant group differences emerged for age (*p* = 0.576) or habitual sleep duration on weekdays (*p* = 0.648) and weekends (*p* = 0.734), with small or negligible effect sizes (all Cohen’s d < 0.30). Detailed results of the group comparisons are shown in Table 2.

**Table 2:** ADHD-traits positive compared to ADHD-traits negative

	Group		Statistics	
Characteristic	ADHD-negative	ADHD-positive	*p*-value	Effect size
n	117 (89.3%)	14 (10.7%)		
Age, years	20.43 (2.37)	20.07 (1.07)	0.576	0.16
*Gender, n* (%)				
Female	114 (97.4%)	13 (92.9%)	0.367	0.08
Male	3 (2.6%)	1 (7.1%)		
BMI, kg/m^2^	20.05 (1.90)	20.07 (1.95)	0.972	0.01
Sleep time (weekday), hours	6.11 (0.95)	5.98 (1.27)	0.648	0.13
Sleep time (weekend), hours	7.89 (1.40)	8.04 (2.03)	0.734	0.10
Chronotype (MSFsc), decimal hours	4.13 (0.78)	4.72 (0.71)	0.014	0.76
*Chronotype category, n* (%)				
Morning type	55 (47.4%)	3 (21.4%)	0.002	0.76
Intermediate type	47 (40.5%)	4 (28.6%)		
Evening type	14 (12.1%)	7 (50.0%)		
Social jetlag, hours	0.89 (0.59)	1.10 (0.95)	0.449	0.33
AIS total score	3.73 (2.72)	5.64 (2.47)	0.013	0.71
*AIS classification, n* (%)				
Minimal (0–5)	67 (57.3%)	3 (21.4%)	0.032	0.23
Mild (6–9)	26 (22.2%)	5 (35.7%)		
Moderate (10–15)	21 (17.9%)	5 (35.7%)		
Severe (16–24)	3 (2.6%)	1 (7.1%)		
PHQ-9 total score	4.38 (3.32)	8.07 (4.68)	< 0.001	1.06
*PHQ-9 classification, n* (%)				
Minimal (0–4)	63 (53.8%)	5 (35.7%)	0.002	0.38
Mild (5–9)	47 (40.2%)	3 (21.4%)		
Moderate (10–14)	6 (5.1%)	5 (35.7%)		
Moderate to severe (15–19)	1 (0.9%)	1 (7.1%)		
Severe (20–27)	0	0		

Values are presented as mean (SD) or n (%). *p*-values are based on independent-samples t-tests (for continuous variables) and Fisher exact tests (for categorical variables)

ADHD traits status was classified using ASRS v1.1 (≥ 14). BMI: Body Mass Index; MSFsc: Mid-Sleep on Free Days corrected; PHQ-9: Patient Health Questionnaire-9; ASRS: Adult ADHD Self-Report Scale v1.1; AIS: Athens Insomnia Scale

### Correlations and regression analysis

ADHD trait scores showed a weak positive correlation with insomnia symptoms (r = 0.29, *p* < 0.001) and a moderate positive correlation with depressive symptoms (r = 0.44, *p* < 0.001), while insomnia and depression showed a moderate positive correlation (r = 0.54, *p* < 0.001) (Table 3). When corrected for measurement unreliability using the Spearman disattenuation formula, these correlations rose to 0.42, 0.61, and 0.72, respectively, indicating that the observed associations are conservative estimates of the underlying constructs.

**Table 3:** Pearson correlations between ADHD traits, insomnia, and depressive symptoms (n = 131)

	ASRS	PHQ9	AIS
ASRS	–		
PHQ9	0.44**	–	
AIS	0.29**	0.54**	–

PHQ-9: Patient Health Questionnaire-9, ASRS v1.1: Adult ADHD Self-Report Scale version 1.1, AIS: Athens Insomnia Scale. **p < 0.001

A multiple linear regression was performed to examine whether ADHD traits independently predicted depressive symptoms after adjusting for relevant covariates. The overall model was significant, F(8, 107) = 5.92, *p* < 0.001, explaining 30.7% of the variance in PHQ-9 scores (R^2^ = 0.31). ADHD trait severity remained a significant positive predictor of depressive symptoms (β = 0.37, B = 0.41, SE = 0.10, *p* < 0.001), even after adjusting for age, sex, BMI, weekday and weekend sleep timing, chronotype (MSFsc), and social jetlag. None of the covariates reached statistical significance, although chronotype and sex approached significance (*p* = 0.055 and 0.059, respectively).

### Mediation analysis

**Fig. 1:**
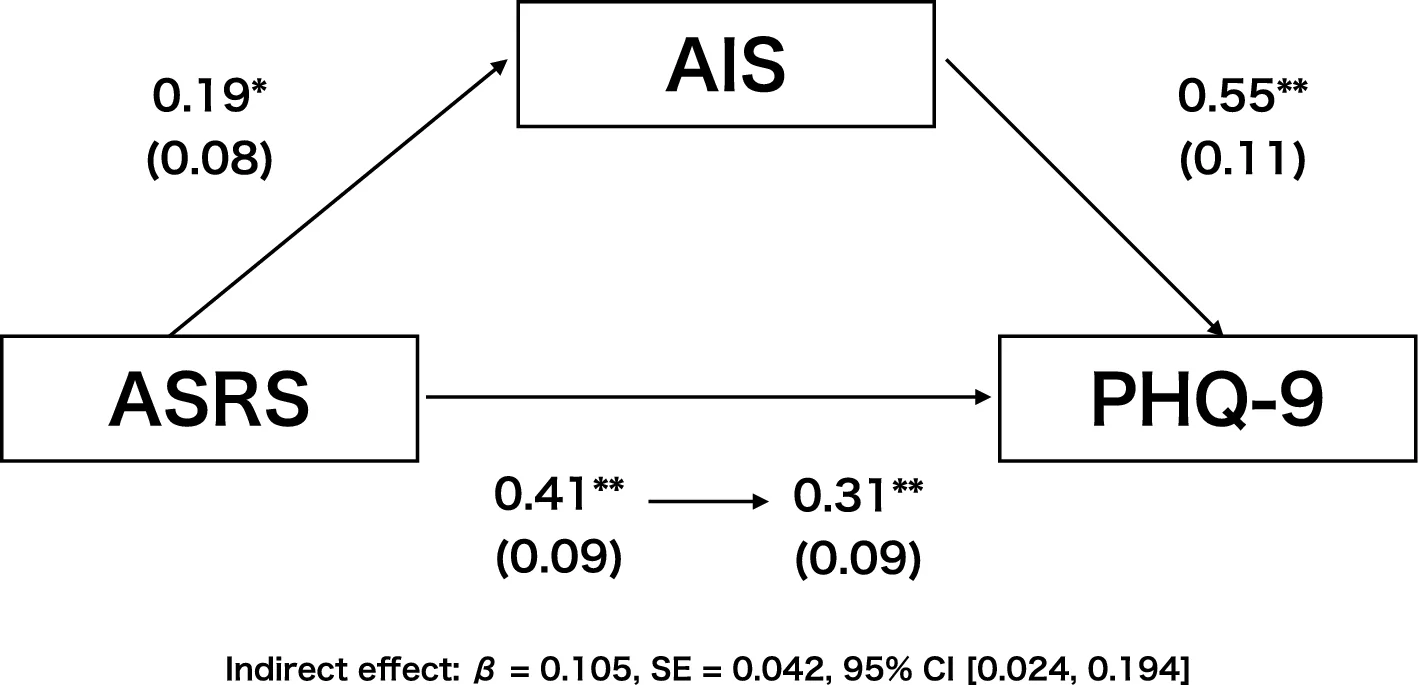
Mediation model testing the indirect effect of ADHD traits (ASRS total score) on depressive symptoms (PHQ-9 total score) through insomnia severity (AIS total score) Standardized regression coefficients are shown with standard errors in parentheses All paths were adjusted for age, sex, BMI, sleep/circadian factors (sleep duration/timing, chronotype, social jetlag). The indirect effect was significant (0.105, SE = 0.042; 95% CI [0.024, 0.194]) *p < 0.05, **p < 0.001

Figure 1 summarizes the bias-corrected bootstrapped mediation model (5,000 resamples; n = 129 after listwise deletion of cases with missing covariate data). ADHD traits significantly predicted insomnia severity (path a: β = 0.253, SE = 0.091, *p* = 0.005), which in turn significantly predicted depressive symptoms (path b: β = 0.416, SE = 0.075, *p* < 0.001). The total effect of ADHD traits on depressive symptoms was significant (c: β = 0.398, SE = 0.083, *p* < 0.001). When insomnia was included as a mediator, the direct effect remained significant but was attenuated (c′: β = 0.293, SE = 0.077, *p* < 0.001).

The indirect effect via insomnia was β = 0.105 (SE = 0.042), with a 95% bias-corrected bootstrap confidence interval of [0.024, 0.194]; Sobel z = 2.49, *p* = 0.013, confirming a statistically significant mediation. This indirect pathway accounted for approximately 26% of the total effect of ADHD traits on depressive symptoms. The findings remained robust after adjusting for age, sex, weekday and weekend sleep duration, chronotype, and social jetlag.

### Sensitivity analyses

To verify that the mediation result did not depend on a single measurement choice, we re-estimated the model with three alternative ASRS specifications. Using the dichotomous (0/1) endorsement scoring, the indirect effect was β = 0.090 (95% CI [0.000, 0.175]; Sobel *p* = 0.039); using the inattention subscale alone, β = 0.089 (95% CI [− 0.006, 0.185]; Sobel *p* = 0.042); and using the primary model with mean-imputed BMI as an additional covariate, β = 0.104 (95% CI [0.014, 0.199]; Sobel *p* = 0.013). Across all four models, the indirect effect was essentially identical in magnitude (β between 0.089 and 0.105) and was statistically significant by the Sobel test, supporting the robustness of the primary conclusion.

## Discussion

This study found that ADHD traits were positively associated with both depressive symptoms and insomnia in Japanese nursing students, and that insomnia severity statistically mediated the cross-sectional association between ADHD traits and depressive symptoms, accounting for approximately one quarter of the total effect. These findings, interpreted within the constraints of a cross-sectional design, identify insomnia as a candidate intervention target. They are particularly relevant in the context of nursing education: compared with students in other fields, nursing students experience higher stress and anxiety, especially in the early stages of training when academic and clinical demands peak [12]. Stressors such as fear of making mistakes or lack of confidence may intensify the impact of ADHD traits on sleep and mood [22].

Given that nursing students are future healthcare providers, their mental health has long-term implications for both educational outcomes and patient care. Although the prevalence of ADHD in nursing students is not well established, previous studies in medical students have reported 1.54% with probable ADHD and 6.91% with elevated screening scores [23]. In the present study, 10.7% screened positive on the ASRS, which likely reflects the difference between screening and diagnostic instruments rather than a true elevation in prevalence.

Our results align with prior research linking ADHD traits, sleep disturbance, and depression. The hyperactive and arousal-dysregulated features of ADHD may disrupt the body clock that regulates the sleep–wake cycle [24, 25]. The present study extends this literature by quantifying insomnia as a partial statistical mediator in pre-licensure nursing students, highlighting the potential of sleep-focused strategies to reduce depressive symptoms in students with ADHD traits. This is consistent with a transdiagnostic perspective in which shared neurobiological mechanisms underpin ADHD, depression, and insomnia [26].

These findings underscore the importance of early support for students with ADHD traits, who may be especially vulnerable to sleep disruption and emotional distress. Educators and student-support staff should regard sleep quality as a modifiable factor when assisting students with neurodevelopmental challenges. Clinical educators are well placed to recognize signs such as fatigue, irritability, or difficulty with early shifts, particularly among students with delayed chronotypes, and to interpret these as possible indicators of mental health needs rather than poor motivation.

Brief screening for sleep problems and referral to targeted, evidence-informed interventions (e.g., sleep-hygiene education, cognitive behavioral therapy for insomnia [CBT-I]) may help reduce depressive symptoms. Practical steps include embedding brief sleep-hygiene education into student orientations [27], establishing referral pathways to CBT-I or mindfulness-based interventions [28], and encouraging open dialogue about workload and stress. Educators should also consider the scheduling needs of students with circadian-rhythm vulnerabilities. Integrating brief, validated sleep and mood screening tools into academic advising may enable early identification and timely support [11]. From a higher-education systems perspective, this preventive approach can be implemented pragmatically within existing student-support structures and may be adaptable across different educational contexts, aligning with institutional efforts to embed mental-health promotion in higher-education ecosystems.

### Bidirectionality and the limits of cross-sectional mediation

Because the design is cross-sectional, the term “mediation” here refers strictly to a statistical decomposition of variance and not to a temporal or causal sequence. Reverse and bidirectional pathways are equally consistent with the present data. Depressive symptoms can disrupt sleep continuity, and chronic insomnia may, in turn, amplify the subjective experience of inattention and impulsivity, thereby inflating self-reported ADHD traits. Reciprocal and feedback-loop models, in which all three constructs sustain one another over time, also remain plausible. Disentangling these alternatives will require longitudinal cohorts with at least three measurement waves, ideally complemented by experimental manipulation of sleep (for example, brief sleep-extension or CBT-I trials) to test causal sufficiency.

### Limitations

This study has several limitations. First, the cross-sectional design precludes any causal interpretation; the temporal ordering implied by the mediation framework cannot be verified, and bidirectional and reciprocal pathways cannot be ruled out. Second, all measures were self-reported, which may have introduced common-method bias and recall bias. Third, the sample was drawn from Japanese nursing students at two universities and may not generalize to other populations or educational systems. Because 96.9% of the sample identified as female, our findings primarily reflect the experience of women in Japanese nursing programs. Sex differences are clinically relevant: in adult ADHD, women more often present with inattentive symptoms and show higher rates of comorbid depression and insomnia than men, and sleep architecture and circadian phase also differ by sex [25]. The number of male participants (n = 4) was insufficient to support a sex-stratified mediation model. Generalization to predominantly male cohorts, to non-Japanese educational systems, or to non-nursing student populations therefore requires replication in more sex-balanced and culturally diverse samples.

Fourth, although the post-hoc Monte Carlo power analysis indicated adequate power (0.82) for the primary indirect effect, power for individual covariate paths was lower; non-significant covariate effects should therefore be interpreted with caution. Fifth, internal consistency for the ASRS-6 was modest (α = 0.677), as is typical for short multidimensional screeners; sensitivity analyses and disattenuated correlations indicated that any residual measurement error is biasing our estimates downwards, so the reported indirect effect should be regarded as conservative. Sixth, unmeasured confounders such as socioeconomic status, anxiety, and academic load may have influenced the results. Future longitudinal and experimental studies are needed to establish temporal precedence and to evaluate the effectiveness of sleep-focused interventions in this population.

## Conclusions

In a pre-pandemic sample of Japanese nursing students, insomnia partially mediated the cross-sectional association between ADHD traits and depressive symptoms, accounting for approximately one quarter of the total effect. This indirect pathway was robust across alternative measurement specifications and adequately powered. While the cross-sectional design precludes causal inference, the convergence of findings suggests that addressing sleep problems may be a promising avenue for supporting the mental health of nursing students with ADHD traits. Educational institutions should consider integrating brief sleep screening and sleep-focused interventions into routine student support systems to strengthen mental health and clinical training readiness, while longitudinal studies test the temporal dynamics of these associations.

## Data Availability

The datasets generated and analyzed during the current study are available from the corresponding author on reasonable request. The data are not publicly available due to participant confidentiality and ethical restrictions.
